# Psychosocial determinants of recidivism risk among incarcerated individuals with a history of substance use: A systematic review

**DOI:** 10.1371/journal.pone.0327810

**Published:** 2025-07-11

**Authors:** Kalaivanan Syasyila, Mohammad Rahim Kamaluddin, Hilwa Abdullah @ Mohd Nor, Paramjit Singh Jamir Singh

**Affiliations:** 1 Centre for Research in Psychology and Human Well-Being, Universiti Kebangsaan Malaysia, Bangi, Selangor, Malaysia; 2 School of Social Sciences, Universiti Sains Malaysia, Penang, Malaysia; University of North Carolina at Chapel Hill, UNITED STATES OF AMERICA

## Abstract

The prevalence of recidivism among individuals with a history of substance use and incarceration remains a significant concern for criminal justice systems worldwide. With significant social and economic ramifications, preventing people with a history of substance use from reoffending is a substantial issue. It is imperative to comprehend the complex connections between psychological and social factors to continue developing successful therapies. To better understand this complexity, this review provides a comprehensive analysis of how psychological vulnerabilities and social barriers combine to influence reoffending. Our search strategies in major databases, including Scopus, Web of Science (WoS), ScienceDirect, PubMed, and PubMed Central (PMC), yielded 34,766 results, which decreased to 858 articles after eliminating ineligible records, duplicates, and records unrelated to the language. Finally, 30 articles qualified to be included in the systematic review following PRISMA guidelines. In addition to social determinants, including family support, community impact, stigma, and peer influence, the review concentrated on psychological issues like drug abuse, dual diagnoses, and early-life adversity. The review highlights the importance of social support, with strong family and community relationships associated with reduced recidivism risk. Mental health issues, particularly those co-occurring with substance use, impede reintegration, with emotional dysregulation and poor decision-making contributing to continued criminal conduct. Other significant risk factors include involvement in a gang, trauma exposure, and a lack of rehabilitative assistance. The results highlight that recidivism is a fundamentally psychological and societal problem rather than just an individual one. Breaking reoffending cycles requires addressing these elements through comprehensive, long-term interventions. Given possible gender disparities in recidivism pathways, future systematic reviews should consider doing separate syntheses for men and women to provide more focused insights.

## Introduction

The term “recidivism” describes the propensity of those who have previously served time in prison to commit new crimes, usually leading to their re-entry into the criminal justice system [1]. This remains a continuous concern in worldwide criminal justice systems, threatening society’s safety and burdening resources dedicated to law enforcement, court procedures, and correctional institutions [2]. Individuals with substance use histories continue to experience disturbingly high recidivism rates. According to a review of data from 33 countries, two-year reconviction rates for regularly reported offense types, including drug-related offenses, ranged from 18% to 55% [3]. In this regard, there is growing evidence that psychosocial variables play a major role in reoffending among people with substance use issues [4,5]. In other words, recidivism is especially imperative in the subset of individuals with a history of substance use and incarceration because of the interconnected dynamics of addiction, criminal activity, and rehabilitation efforts [6].

Those who commit crimes involving the use of substances, such as drug possession, distribution, or offenses committed under the influence of substances (i.e., drug-fueled crimes), are considered individuals with a history of substance use and incarceration in this study. According to research by Zgoba et al. [7], which was carried out in the United States of America (USA) and was mostly made up of men (93.0%), drug use offenses were among the most common, accounting for 25.6% of incarceration offenses. Individuals with a history of substance use have significantly higher recidivism tendency for violent crimes (i.e., crimes against people, including attempted, completed, and aggravated forms of murder, manslaughter, unlawful threats, harassment, robbery, arson, assault on an official, kidnapping, stalking, coercion, and all sexual offenses except the purchase of sexual services) than those convicted of non-violent crimes, with many cycling in and out of prison numerous times in their lives [8]. To further highlight the complexity of this issue, Mitchell et al. [9] suggested that while the majority (88%) of incarcerated individuals in their sample had a substance use problem, virtually all (98%) had at least one diagnosed mental condition related to substance use. This strengthens the documented link between substance use and mental illnesses, since those with serious mental illnesses may be more susceptible to using substances as a kind of self-medication [10]. This implies that although the use of substance is an important factor, recidivism is also influenced by other psychological and social risks.

These statistics demonstrate the high prevalence of substance-related crimes among incarcerated people. Substance use increases the possibility of reoffending after release, even among those who do not fit the criteria for substance use disorder (SUD) [11]. According to research, substance use, whether it be chronic or sporadic, can influence illicit behavior by enhancing impulsivity, impairing judgment, and perpetuating cycles of imprisonment and relapse [12]. Furthermore, post-release substance usage has been associated with problems in social reintegration, raising the probability of recidivism [13]. As a result, the relationship between addiction and criminal activity often prolongs recidivism cycles, therefore complicating treatment and successful community reintegration [14].

Moreover, psychosocial factors are important in understanding recidivism among those who have used substances in the past. Agnew [15] first presented the General Strain Theory (GST), which has now been well supported in research focusing on the relationship between strain, negative emotions, and criminal conduct. According to a recent study, persistent criminal behaviors are highly influenced by socioeconomic instability, social marginalization, and unfavorable childhood experiences, especially for those with a history of substance use [16]. These pressures produce a vicious cycle of helplessness, rage, and frustration that raises the risk of maladaptive coping mechanisms, including substance use and criminal activity.

In parallel, the self-medication theory [10] indicates that individuals do not take substances for recreational purposes, but rather to cope with underlying psychological distress. According to recent empirical research, individuals who have experienced trauma, psychological disorders, or ongoing stresses are more prone to turn to substance use as a coping strategy [17]. The claim that recidivism among individuals with a history of substance use is caused by unresolved psychological distress and social disadvantages that prolong reoffending cycles rather than just criminal intent is supported by this [18].

According to Bandura’s [19] Social Learning Theory (SLT), people pick up new behaviors from the people around them through imitation, reinforcement, and observation. This implies that in the context of recidivism, people who have a history of substance use may carry on committing crimes if they are frequently exposed to peers who are deviant or to settings that normalize and encourage such conduct [20]. For instance, those who are released from prison could rejoin groups that encourage substance use and criminal activities, which would make them more likely to commit crimes again due to the nature of groupthink [13]. Furthermore, these habits can be further solidified by rewards, such as peer praise, financial gain, or avoiding withdrawal symptoms, which makes it more challenging to refrain from crime [21]. These findings highlight the continued importance of SLT in understanding the function of social contexts in affecting recidivism risk among incarcerated individuals with histories of substance use.

Although previous studies addressed various kinds of societal and psychological factors linked to individuals with substance use and recidivism, the results have been inconclusive. Many studies fail to adopt a more comprehensive psychosocial framework, instead concentrating on merely certain factors, including early experiences or social opportunities [22–27]. For instance, studies on psychological factors typically concentrate on discrete factors such as mental health conditions or cognitive functioning [25,28], but they often fail to consider these variables in the context of larger social frameworks, such as family support or community reintegration initiatives, which causes them to miss the bigger picture. The studies that have already been conducted also frequently include general criminal populations without separating individuals with substance-using histories, who have distinct risk profiles and rehabilitation needs. To date, there has been no thorough, systematic synthesis that particularly consolidates the psychosocial determinants that influence recidivism risk in this population. Addressing this gap is critical for informing more focused treatments and policy measures suited to the needs of individuals with a history of substance use and imprisonment.

It is imperative to integrate current research on psychosocial factors that influence recidivism to progress theoretical knowledge as well as real-world applications in correctional systems and rehabilitation efforts. Although studies suggest that men and women may face distinct imprisonment trajectories and sentence durations [29], the objective of this research is to uncover the psychosocial risks of recidivism that are relevant to all demographic groups. Synthesizing only a few studies that give gender-disaggregated findings would lead to an incomplete and perhaps biased portrayal of the larger body of information. Therefore, our goal is to offer thorough insights that can inform interventions that assist individuals with a history of substance use and imprisonment by emphasizing universal factors. Such synthesis is critical for establishing targeted interventions that address the complex nature of recidivism among individuals with a history of substance use and incarceration. Hence, this review seeks to reveal underlying patterns and associations that may not be readily apparent in individual research by combining and evaluating information from several fields. The purpose of this review is to explore the social and psychological elements that influence the risk of recidivism among the incarcerated population with a history of substance use. It also investigates how these two realms interact, noting that psychological and social factors are frequently connected rather than isolated. In this context, psychological elements relate to intrapersonal problems at the individual level, social variables include more general relational and structural dynamics within an individual’s surroundings.

## Methods

This review has been registered under PROSPERO (CRD420251007526) and followed the Preferred Reporting Items for Systematic Reviews and Meta-analyses (PRISMA) guidelines [30]. This review focuses on identifying the psychosocial determinants of recidivism risk among incarcerated individuals with a history of substance use, rather than determining the strongest or most consistent predictors. While quantitative data (e.g., odds ratios, coefficients) from the included studies are presented in Table 2, the primary aim of this review is to synthesize the direction and nature of associations between these factors and recidivism, rather than to conduct a direct comparison of effect sizes or to rank predictors by strength. The identification, eligibility, screening, and inclusion phases made up the four stages of this systematic review’s analysis. A thorough description of the review protocol is provided in S1 Appendix.

**Table 1:** Inclusion and exclusion criteria.

Criteria	Inclusion	Exclusion
Type of Study	Studies published between 2004 and 2024	Non-primary research articles (e.g., reviews, letters to the editor, minutes of meetings, or informative notes)
	Empirical research articles presenting primary data	
	Articles written in English (Malay was also considered, but no studies in Malay were found during the search)	
	Empirical research articles presenting primary data	
Population	Participants with a history of substance use and recidivism, including those currently incarcerated, formerly incarcerated, or recently released.	Participants who are first-time incarcerated individuals
Outcome	Studies that specifically examine risk factors associated with recidivism among individuals with a history of substance use	

**Table 2:** General description of the study.

Authors	Country	Nature of Study	Sample Size & Study Population	Follow-up Time	Rate of Recidivism	Measures/ Data
Mohd Alif et al. (2024) [52]	Malaysia	Qualitative research through purposive and snowball sampling	30 homeless former prisoners (24 males, 6 females), nine Malaysian government agency officers, and nine volunteers	3 years	There were 30,902 repeat offenders out of 210,251 persons who were released.	A robust interview protocol and a semi-structured form
Ojansuu et al. (2023) [40]	Finland	Quantitative research; cohort study of 5, 10, 15 years	501 offenders who were released from forensic psychiatric care (434 males; 67 females)	5-15 years	There were 83 patients (16.6%) who had committed a recidivistic offense, 48 (9.6%) of which were violent.	Database from National Institute for Health and Welfare (THL) and Institute of Criminology and Legal Policy
Karlsson and Håkansson (2022) [4]	Sweden	Quantitative research; retrospective cohort study	4,207 prisoners (3,786 males; 421 females)	2.7 years	68% committed a second offense.	Addiction Severity Index (ASI)
Orlando and Farrington (2021) [56]	Argentina	Quantitative research; cross-sectional, interview-based quantitative study	124 young offenders	2 years	3.1% of juvenile offenders are female recidivists and 96.9% of young offenders are male.	Database of National Supreme Court of Argentina (NSCA); ofence history, family factors, educational factors, substance use history factors, social factors, community and societal infuences
Molina-Coloma et al. (2021) [53]	Spain	Quantitative research; case-control study with a non-probability convenience sample	40 recidivists (20 males; 20 females)	1 year and above	Of the sample, 28% had illegally obtained drugs, and 48% had committed crimes against property.	Questionnaire of sociodemographic characteristics, Questionnaire of crime characteristics, Millon Multiaxial Clinical Inventory-III (MCMI-III), Barratt Impulsiveness Scale (BIS-11), and Buss-Perry Aggression Questionnaire (BPAQ)
Wallace and Wang (2020) [60]	USA	Quantitative research; longitudinal study	871 male individuals who have committed serious and violent offenses	3–15 months	There is a 44% reduction in the likelihood of recidivating for every increase in an individual’s mental health after release compared to their health while incarcerated.	SVORI, Short-Form Health Survey (SF-12)
Link et al. (2019) [50]	USA	Quantitative research; longitudinal study; four waves (30 days prior to their release from institutional corrections and 3, 9, and 15 months post release)	1,697 men with extensive criminal histories	3–15 months	At 3 months, 58% were successfully re-interviewed; 61% were interviewed at 9 months, and 66% at 15 months.	Physical health limitations, Center for Epidemiologic Studies – Depression scale, Family conflict – self-measured, Employment status – self-measured, Financial problems – self-measured
Glover (2018) [47]	Ghana	Qualitative research; semi-structured interview guide on purposively selected recidivist	30 recidivists (15 males; 15 females)	8 years	Frequency of armed robbery: 9 (30%), stealing: 11 (37%), defilement: 3 (10%), fraud: 2 (7%), assault: 4 (13%), substance abuse: 1 (3%).	Semi-structured interview guide
Link and Hamilton (2017) [49]	USA	Quantitative research; longitudinal study using cross-lagged panel models	1,697 adult male offenders	3-15 months	At 3 months, 16%, 32% at 9 months; and 30% at 15 months.	Serious and Violent Offender Reentry Initiative (SVORI) evaluation
Skeem and Lowenkamp (2016) [57]	USA	Quantitative research; longitudinal cohort study	34,794 federal offenders	12 months	Violent recidivism was a 72–75% possibility for offenders.	Post Conviction Risk Assessment (PCRA) and data from the National Crime Information Center (NCIC)
Chang et al. (2015) [36]	Sweden	Quantitative research; longitudinal cohort study	47,326 prisoners (43,840 male and 3,486 female prisoners)	10 years	Of the male inmates, 10,884 (or 25%) committed violent crimes again. 379 female inmates (11%) committed violent offenses again.	Data obtained from both inpatient and outpatient registers, and other population-based registers
Begun et al. (2015) [33]	USA	Quantitative research; short-term, longitudinal study. Time 1 = in-person, structured interviews Time 2 = follow-up through telephone Time 3 = follow-up interviews in the community.	75 men and 62 women in jail, prison, or community-based correctional facilities (CBCFs)	4 months and above	55% men and 45% women	Mental Health Consumer Outcomes System: Adult Consumer Form
Van der Put et al. (2014) [59]	Netherlands	Quantitative research; cross-sectional secondary data	3,317 male juvenile offenders substance use problems	6 months	Abstain from substance use (ASU): 34.0% Substance-using (SU): 42.3% substance use problems (SUP): 50.1%	Washington State Juvenile Court Assessment (WSJCA)
Barrick et al. (2014) [43]	USA	Quantitative research; inmate surveys were conducted 30 days before release and 3, 9, and 15 months following release, and post-release recidivism was discovered using data from state agencies and the National Criminal Information Center (NCIC).	255 high-risk female offenders with extensive criminal and substance use histories	3-15 months	Nearly half (44%) were reincarcerated within 5 years of release.	Global Severity Index (GSI) and self-developed questionnaire to assess family emotional support
Stahler et al. (2013) [41]	USA	Quantitative research; retrospective follow-up study	5,354 prisoners (234 males; 5,120 females)	3 years	35% of the sample were reincarcerated within 3 years.	TCU Drug Screen, data from Bureau of the Census
Mulder et al. (2012) [55]	Netherlands	Quantitative research; retrospective cohort study	728 male juvenile offenders	2 years	Serious violent: 69%, violent and property: 89%, property: 82%, sex offenders: 47%	FPJ and the Official Judicial Offence Registry of the Netherlands supplied offence data
Wilson et al. (2011) [42]	USA	Quantitative research; retrospective cohort study	20,112 offenders admitted to jail	4 years	50% had at least one readmission after their initial release.	Dataset from Medicaid records (Philadelphia behavioral health system) and Philadelphia County jail system records
Tewksbury et al. (2011) [58]	USA	Quantitative research; longitudinal study	248 male offenders	1 year	A relatively small (9.7%) portion of the sample recidivated at a very low rate.	Data from the New Jersey State Police Computerized Criminal History System, National Crime Information Center’s Interstate Identification Unit, New Jersey Department of Corrections’ (NJDOC) Offender-Based Correctional Information System (OBCIS)
Luther et al. (2011) [51]	USA	Qualitative research; focus groups	51 returning prisoners (22 males; 29 females)	1 year	Within the first six months after their reintegration, 30% of ex-offenders are arrested again, and 44% do so within a year of their release.	Structured interview questions were developed by the authors
Mulder et al. (2010) [54]	Netherlands	Quantitative research; longitudinal study with retrospective cohort approach	728 male juvenile offenders	2 years	32.9% of the delinquents in the study recidivated.	Juvenile Forensic Profile (FPJ); (Child Behavior CheckList, Structured Assessment of Violence Risk in Youth, Psychopathy Checklist–Youth Version, Juvenile Sex Offender Assessment Protocol, HCR-20 Violence Risk Assessment Scheme)
Grunwald et al. (2010) [44]	USA	Quantitative research; retrospective cohort study	7,061 delinquent male juveniles	13 months	40% of young offenders reoffended, 14% with a drug crime, 10% with a violent crime, and 11% with a property violation. Only 5% of offenders had a sexual crime.	Secondary analysis from the Program Development and Evaluation System (ProDES) and neighborhood survey data Philadelphia Health Management Corporation (PHMC)
Castillo et al. (2010) [35]	USA	Quantitative research; retrospective comparative cohort study.	307 adult offenders (175 male; 132 female)	12 months and above	The study found a recidivism rate of 68% among high-risk offenders.	Secondary data from case files and program records
Benner et al. (2010) [34]	USA	Quantitative research; secondary analysis of existing mental health and juvenile justice data was conducted.	761 juvenile offenders (469 males, 292 females)	4 years	Children who had experienced abuse were more likely to be arrested as adults (42%), compared to 27% as juveniles.	Massachusetts Youth Screening Instrument-Version 2 (MAYSI-2); The Thought Disturbance subscale
Ferguson et al. (2009) [46]	Australia	Quantitative research; retrospective study	208 mentally ill offenders (157 male; 51 female)	5 years	More than half of the patients (51.0%) reoffended.	The Level of Service Inventory–Revised: Screening Version (LSI-R:SV)
LeBel et al. (2008) [5]	USA	Quantitative research; longitudinal study	126 male property offenders	10 years	During the 10-year follow-up period, 23 men (18%) were able to avoid being convicted again.	Self-report interview
Huebner et al. (2007) [48]	USA	Quantitative research; retrospective follow-up study	322 men aged 17–24 years released from prison in a Midwestern state	9 years	During the research period, 37% of the sample was reconvicted: 15% for violent, personal crimes, 32% for property crimes, 24% for drug crimes, 12% for weapons-related offenses, and 17% for other offenses.	Inmate surveys, presentence investigation (PSI) reports, and official reports maintained in a central data repository
Kubrin and Stewart (2006) [45]	USA	Quantitative research; observational cohort study	4,630 former inmates (3,473 males; 1,157 females)	12 months	28% of the sample was rearrested within the study period.	Index of Concentration at the Extremes (ICE)
Hepburn (2005) [38]	USA	Quantitative research; longitudinal study	4,479 drug use offenders (3,673 males; 806 females)	5 years	During the follow-up surveillance period, 43% of offenders were apprehended again.	Observation by the Office of the County Attorney in Maricopa County (Phoenix)
Långström et al. (2004) [39]	Sweden	Quantitative research; retrospective follow-up study	1,215 adult male offenders	5 years	5.9% for sexual (as defined above), 10.4% for violent nonsexual (i.e., attempted or completed homicide, assault, or robbery), and 15.1% for any violent (sexual or violent nonsexual) recidivism.	International Classification of Diseases (ICD-9) and (ICD-10)
Gagliardi et al. (2004) [37]	USA	Quantitative research; cohort study	333 inmates (233 male; 100 female)	27–55 months	Recidivism rates for general population (GP) offenders were projected to be lower since 2% of the MIO’s felony recidivism data was not available to them. Compared to 41% of MIOs, 38% of GP offenders were found guilty of a new offense.	Washington State Institute for Public Policy (WSIPP) databases for Washington records and the National Crime Information Center (NCIC) databases.

### Information sources

The databases Scopus, Web of Science (WoS), ScienceDirect, PubMed, and PubMed Central (PMC) were used to find relevant articles on the risk of recidivism among substance-using individuals for this systematic review. The selection of these databases was based on their extensive coverage of peer-reviewed literature in several fields, ensuring a complete and interdisciplinary review process. Following the PRISMA guidelines, the search strategy was rigorously constructed to retrieve findings published between 2004 and 2024. To include the most recently published and relevant research on recidivism and substance use, a 20-year timeframe was selected that reflected current trends and theoretical developments in the area. The study attempts to give a current synthesis of the psychosocial factors of recidivism among people with a history of substance use and imprisonment by concentrating on these particular databases and periods.

### Search strategy

The search method was created especially to find studies on the risk of recidivism among substance-using individuals. This strategy comprised a variety of search phrases and Boolean operators with regard to the psychological and social elements that impact recidivism. The search strategy includes broad but pertinent phrases such as ‘psychological factors,’ ‘social factors,’ and ‘recidivism risk.’ This method was used to prevent inadvertently limiting the scope and to include research that looked at various psychosocial influences, even if they employed different terminologies. Following data extraction and synthesis, specific variables, including social support, mental health, and peer influence, were discovered. Our search strategy targeted abstracts, keywords, and, where supported by the database, full text (e.g., ScienceDirect, PMC). In databases such as WoS and PubMed, searches were limited to abstracts and keywords. The following search expression was used, with adaptations to meet each database’s requirements: (“psychological factors” OR “psychosocial factors” OR “mental health” OR “psychiatric disorders” OR “psychological disorders”) AND (“social factors” OR “social environment” OR “socioeconomic status” OR “social support” OR “family support”) AND (“recidivism” OR “reoffending” OR “criminal recidivism” OR “drug offenders” OR “substance abuse”) AND (“risk factors” OR “risk”).

### Eligibility and screening

The data collection approach for this systematic review was rigorously planned to ensure that significant data were extracted comprehensively and accurately from the included studies. Several steps were taken to screen the literature using Covidence software. These procedures were aided by the establishment of inclusion and exclusion criteria, which ensure that a rational selection of papers is selected for review. These criteria were adapted to ensure that the most relevant research on the psychosocial causes of recidivism among substance users was chosen. This allowed the researcher to precisely achieve the study’s objective. The final criteria, shown in Table 1, represent a thorough and methodologically sound assessment procedure.

**Fig 1:**
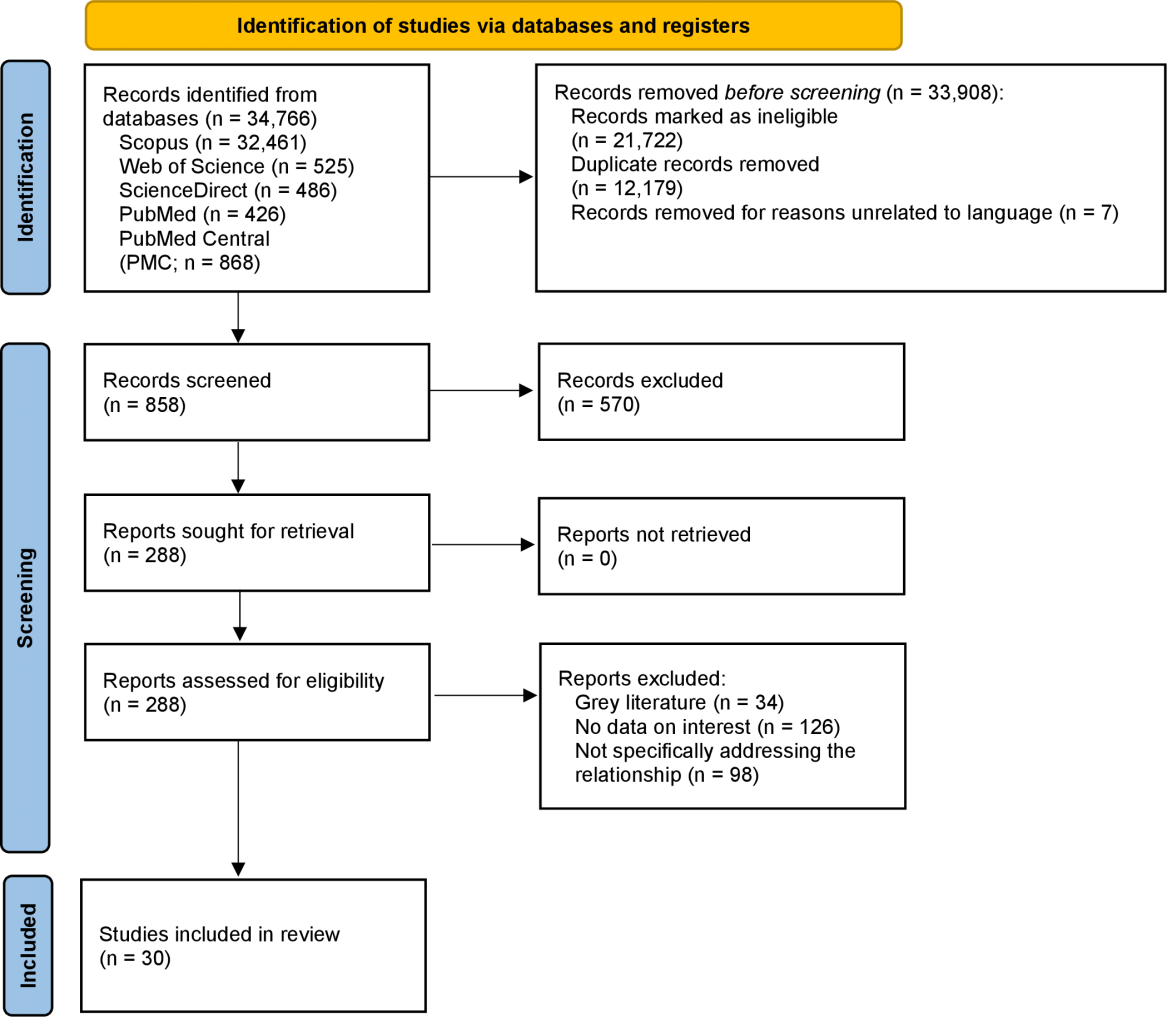
PRISMA Flowchart.

The Covidence software program was used to gather all recognized records from the database searches. The integrated feature of Covidence was used to automatically identify and eliminate duplicates. A total of 34,766 records had been identified across several databases during the initial search on July 11, 2024. Prior to screening, 33,908 records were eliminated, of which 21,722 were deemed ineligible, 12,179 were duplicates, and 7 were eliminated for non-linguistic reasons. 858 records were evaluated in the screening phase; 570 of those were discarded because they were not full-text or did not have open access. 288 reports were then assessed for eligibility. Of these, 34 were deemed to be grey literature, 126 were found to lack pertinent data, and 98 did not explicitly address the relationship of interest. Finally, 30 papers matched the pre-established criteria and were included in the analysis. Fig 1 shows the PRISMA Flowchart that illustrates this selection procedure in detail.

### Quality assessment

To ensure the validity and reliability of the findings presented in this systematic review, we used rigorous methodologies to analyze the risk of bias in the included studies. The evaluation was carried out using Covidence. Covidence enables numerous reviewers to conduct independent assessments, offering a platform for rapid and reliable data extraction and risk of bias examination.

To ensure impartiality and reduce the possibility of reviewer bias, each study in this review was critically evaluated for bias by two reviewers working separately. Reviewers assessed the studies according to predetermined standards, paying particular attention to participant selection, research design, and outcome. If there were disagreements amongst the reviewers, they were resolved by discussion or, if required, consultation with a third reviewer.

To assess the methodological quality of the studies further, we used the Mixed Methods Appraisal tool [31] (see Supplementary Material 1). MMAT is especially used to appraise the quality of studies using diverse research approaches, such as qualitative, quantitative, and mixed methods. Two independent reviewers evaluated each study, and disagreements were resolved through discussion. The agreement degree between reviewers was assessed through Cohen’s Kappa (K). The observed value (K = 0.86) was considered almost perfect [32]. We were able to ensure that the findings were supported by solid and well-conducted research by using this technique to appraise the quality of the included papers carefully. Overall, the risk of bias was minimal in the included research since most followed rigorous methodological criteria.

### Outcome measure

The key outcome of interest in this review was recidivism, which is often measured using administrative database records of rearrest, reconviction, or reincarceration. The primary objective, however, was to assess and synthesize the psychosocial elements that influence recidivism in people with a history of substance use and incarceration. A uniform effect measure was not possible due to the inconsistencies in study designs and recidivism definition. A narrative synthesis approach was used to incorporate information about psychological vulnerabilities, mental health concerns, social support, family dynamics, and structural disadvantages.

## Results

### Description of studies

This evaluation looked at 30 studies on the risk of recidivism among individuals with a history of substance use and incarceration by year of publication, country of origin, nature of study, risk factor type, and length of follow-up to provide a thorough synthesis (see Table 2). The studies were published between 2004 and 2024, with peaks in 2010 (*n* = 4) and 2011 (*n* = 3). Most years provided one study apiece, demonstrating an ongoing research interest across two decades. The majority of the research was done in the United States (*n* = 18), with Sweden and the Netherlands (*n* = 3 each) and Argentina, Australia, Finland, Ghana, Malaysia, and Spain (*n* = 1 each) also represented.

The methodological approach was primarily quantitative (*n* = 27), including retrospective and longitudinal cohort studies, with only three studies using qualitative approaches such as interviews and focus groups.

The gender composition of the samples in the 30 analyzed studies showed a preponderance of male participants. Eleven studies specifically focused on male incarcerated individuals, frequently seen in adult or juvenile populations. However, there was just one study that exclusively examined the female population in prison. The majority of research (*n* = 18) had male and female participants, with varying sample sizes and gender distributions. These mixed-gender samples included large national datasets (i.e., 47,326 prison population, consisting of 43,840 males and 3,486 females) and small-scale studies (i.e., 30 recidivists evenly divided by gender). The populations under study were diverse and included young, incarcerated individuals, adults with mental illnesses or forensic psychiatric conditions, and homeless former incarcerated individuals.

In terms of the types of risk factors investigated (see Table 3), ten studies focused solely on psychological variables [33–42], three assessed social aspects alone [43–45], and 17 studies combined psychological and social dimensions [4,5,46–60]. The period of follow-up utilized to determine recidivism, which spanned from 6 months to 15 years, was a key differentiating feature. A substantial number of studies utilized short- to mid-term follow-ups (e.g., 3–15 months, 1–3 years), which allowed for the evaluation of immediate post-release risks. One study followed individuals for up to 15 years, whereas others used longer tracking periods of 5–10 years.

**Table 3:** Findings of the study.

Authors	Factors	Study Outcomes/Results
Mohd Alif et al. (2024) [52]	Psychological factor – drugs confer self-confidence, drug addiction, mental illness, personal choice, unrepentant, survival Social factor – peer influence, environmental influence, a drug activity-filled environment	Theme: 1. Peer influence 2. Drugs confer self-confidence 3. Drug addiction 4. Mental illness 5. Survival 6. Peer influence 7. Environmental influence 8. Personal choice 9. Unrepentant 10. A drug activity-filled environment 11. Drugs as a necessity Themes reflected both psychological factors, such as addiction, mental illness, and drugs as a source of self-confidence, and social drivers like peer influence, unemployment, and drug-saturated environments, all contributing to continued drug use and criminal behavior.
Ojansuu et al. (2023) [40]	Psychological factor – SUD, mental health treatment	Individuals with SUD were 2.65 times more likely to recidivate, and long. The most significant predictor linked with an increased probability of both general and violent recidivism was a drug use disorder. Extended forensic mental health treatment is linked to decreased overall reoffending rates.
Karlsson and Håkansson (2022) [4]	Psychological factor – suicide attempt, psychiatric illness Social factor – homelessness	Suicide attempt (*HR* 0.95) and psychiatric medication (*HR* 1.2) are psychological factors associated with recidivism, while homelessness (*HR* 1.4) is a key social factor contributing to repeat offences.
Orlando and Farrington (2021) [56]	Psychological factor – substance use Social factor – delinquent peers, ineffective use of leisure time, family conflict, criminality in the family	Recidivism was strongly associated with substance use (92%), as well as key social risk factors including delinquent peers (100%), ineffective use of leisure time (97%), family conflict (91%), criminality in the family (49%). The most significant independent predictors of juveniles’ recidivism were delinquent peers, drug misuse, low academic achievement, poor attendance, and disorganized communities.
Molina-Coloma et al. (2021) [53]	Psychological factor – psychological problems, clinical personality traits, substance and alcohol dependence, aggression Social factor –disciplinary problems with peers in prison	Recidivists were more likely to have prior psychological problems (37.5% vs. 8.16%, χ2(1) = 11.28, p < .001), and exhibited higher rates of antisocial (*d* = 0.89), aggressive-sadistic (*d* = 0.70), and borderline (*d* = 0.62) personality traits, as well as greater substance (*d* = 0.84) and alcohol dependence (*d* = 0.79). Recidivist have increased levels of physical aggressiveness, alcohol and drug use, and antisocial, borderline, and aggressive-sadistic behavior. More crimes against property are committed by recidivists; a higher percentage of them have a history of juvenile delinquency and more disciplinary records in adult prison.
Wallace and Wang (2020) [60]	Psychological factor – mental health issues Social factor – gang involvement during incarceration	Poor in-prison mental health increased recidivism odds by 57% (*OR* = 0.632), and gang involvement during incarceration more than doubled the risk (*OR* = 2.40). Worsening mental health post-incarceration significantly increased recidivism risk, with predicted probabilities rising from 4.6% to 23.0% depending on changes in mental health status. Poor in-prison mental health appears to influence the risk of recidivism overall.
Link et al. (2019) [50]	Psychological factor – depression Social factor – family conflict, financial strain	Depression indirectly increased recidivism through family conflict and financial strain (e.g., depression → family conflict → crime → reincarceration, Std. Estimate = .009; *CI* [.002,.021]) The direct criminogenic impacts of poor family-marital relationships and school-work domains have led to their recognition as key risk factors for recidivism. A major contributing factor to recidivism and relapse is unemployment.
Glover (2018) [47]	Psychological factor – substance abuse and low interest in rehabilitation Social factor – re-integration challenges, cell population, peer influence	Theme: Psychological 1. Association with bad friends 2. Low interest in rehabilitation 3. Substance abuse Theme: Societal 1. Difficulty in re-integration 2. Character contamination – cell population 3. Conduct of some prison officers 4. Not adequately reformed
Link and Hamilton (2017) [49]	Psychological factor – mental health, emotional support Social factor – poor supervision	Higher emotional support (*p* < .01), probation/parole supervision (*p* < .01), and mental health treatment (*OR* = 0.34, *p* < .01) significantly reduced substance use, while mental health issues (*OR* = 1.74, *p* < .01) increased both recidivism and drug use. Reductions in the probability of reporting drug use at three and fifteen months were linked to emotional support from family members. There was a correlation between being under criminal justice supervision (probation or parole) and a lower chance of reporting substance misuse by the 3-month interview and of being arrested again by the 9-month interview. While having a job was associated with a decreased risk of recidivism, it had no discernible effects on drug use. Rearrest outcomes did not exhibit a significant correlation with characteristics related to mental health and mental health services.
Skeem and Lowenkamp (2016) [57]	Psychological factor – substance use Social factor – social networks	Substance use (*OR *= 1.29, 99.9% *CI *= 1.10–1.51, *p *< .001) significantly predicted general recidivism, highlighting a key psychological risk factor. Several social factors were also associated with increased odds of reoffending, including problematic social networks (*OR *= 1.15). Individual histories, environmental factors, and judicial systems all interact to influence recidivism outcomes.
Chang et al. (2015) [36]	Psychological factor – psychiatric disorders, substance use disorder (SUD)	Diagnosed psychiatric disorders were associated with an increased hazard of violent reoff ending in male (adjusted *HR* 1·63 [95% *CI* 1·57–1·70]) and female (2·02 [1·54–2·63]) prisoners, and these associations were independent of measured sociodemographic and criminological factors, and, in men, remained substantial after adjustment for unmeasured familial factors (2·01 [1·66–2·43]).
Begun et al. (2015) [33]	Psychological factor – mental health help-seeking behavior	56.5% of participants anticipated a need for mental health services during reentry, yet only 15% received any, despite 48% reporting the need. This gap between anticipated need and actual receipt of services highlights a psychological risk factor for recidivism, particularly among substance users, as untreated mental health issues may hinder successful reentry. Pre-incarceration drug usage was prevalent among the majority of research participants who had been released from prison. Risky or binge drinking was frequent, as was using other drugs, sometimes as often as twice a week. After being released, over two-thirds of participants expected to require some assistance to deal with alcohol or other substance issues. Nearly 70% of individuals reported having been prescribed medication for mood, behavior, or emotional issues, indicating that mental health problems were also prevalent.
Van der Put et al. (2014) [59]	Psychological factor – aggression, attitudes Social factor – family, relationship difficulties	Higher recidivism odds were linked to elevated risk scores in aggression (*OR* = 1.13), relationships (*OR* = 1.07), attitudes (*OR* = 1.06), and family issues (*OR* = 1.02), with school problems also predictive (*OR* = 1.03). In comparison to ASU teenagers, substance-using individuals, especially those with substance use issues, had higher risk factors and fewer protective factors in the areas of education, free time usage, relationships, family, attitude, aggressiveness, and skills. The ASU group showed greater correlations with recidivism than the SUP group did with the majority of the risk/protective factors. In addition to risk factors, substance use was a distinct predictor of recidivism.
Barrick et al. (2014) [43]	Social factor – family emotional support	Post-release family emotional support was significantly associated with a reduced likelihood of recidivism (*OR* = 0.814, *p* < 0.05). Higher family emotional support was associated with a delay in reincarceration (Coefficient = 0.1013, *p* < 0.05). Having ongoing communication with family members is linked to reduced recidivism rates and increased levels of instrumental and emotional support after release. Emotional support is as significant as having family members truly show support by making an effort to talk on the phone or pay a visit.
Stahler et al. (2013) [41]	Psychological factor – substance use	Substance use increased the likelihood of reincarceration by 39% (*OR* = 1.39) High-risk (“spatially contaminated”) communities, poor thinking and decision-making behaviors, and drug relapse contribute to recidivism among substance users.
Mulder et al. (2012) [55]	Psychological factor – psychological disorders, low impulse control Social factor – parenting skills, criminal behavior in the family, peer rejection, lack of intimate relationships, lack of social skills	Psychological factors (e.g., conduct disorder, substance abuse, anxiety disorder) and social factors (e.g., poor parenting, criminal peers, peer rejection) significantly differ between serious violent offenders (*n* = 114), violent property offenders (*n* = 334), and sex offenders (*n* = 66), with recidivist groups showing higher levels of these risks. In addition to high scores for conduct disorder, low impulse control, alcohol, and drug abuse (including during the offense), involvement with criminal peers, criminal behavior in the family, lack of parenting skills, authority issues, truancy, and antisocial behavior, the sample has risk factors for alcohol abuse, lack of conscience, and problem insight.
Wilson et al. (2011) [42]	Psychological factor – dual diagnoses	Individuals with co-occurring serious mental illness and substance use disorders had the highest 4-year recidivism rate (68%), while those with no diagnosis or only mental illness had the lowest (50%), highlighting substance use as a key psychological risk factor. While 68% of people with dual diagnoses experienced at least one readmission to jail in the four years following their release, they had the greatest rate of readmissions to jail over the research period. One major factor contributing to the recidivism rate of mentally ill individuals released from US urban jails is substance abuse.
Tewksbury et al. (2011) [58]	Psychological factor – substance use Social factor – two-parent household	In a study comparing low- vs. high-risk recidivism trajectories, drug problems (70.5% vs. 42.1%, *p* < .01) and coming from a non-two-parent household (48.8% vs. 70.9%, *p* < .01) are significantly more common among high-risk individuals. Due to a dearth of rehabilitation and support services that effectively address underlying issues, including deviant sexual preferences and poor impulse control, a much higher proportion of individuals with sexual offenses in the high-risk trajectory recidivated for a sex crime after being released from prison.
Luther et al. (2011) [51]	Psychological factor – psychological strain or stress, internalized stigma, lack of emotional support, motivation, or mindset around recovery/reintegration Social factor – family or community support	Themes: 1. Immediate reentry experience 2. Barriers to successful reentry 3. Facilitators to successful reentry 4. Common locations for HIV testing 5. Common locations for substance abuse treatment Psychological challenges during immediate reentry and the role of emotional support as key facilitators, while social factors such as structural barriers to reentry and the accessibility of HIV testing and substance abuse treatment shaped reentry outcomes. Lack of discharge planning led – basic needs such as stable housing and employment not being met.
Mulder et al. (2010) [54]	Psychological factor – psychopathology, negative psychological traits Social factor – criminal environment, social influences	This study identifies both psychological (e.g., conduct disorder, gambling addiction), social (e.g., poor parenting, criminal environment), and substance-related factors (e.g., alcohol addiction, substance abuse) significantly contributing to overall recidivism (62.9%) and violent recidivism (37.1%) among juvenile offenders. The following were identified as risk factors for recidivism: conduct disorder; family risk factors (poor parenting skills, criminal behavior in the family, history of physical and emotional abuse); involvement with criminal peers; and past criminal behavior (number of past offenses, young age at first offense, unknown victim of past offenses). Serious (violent) recidivism was predicted by having an unidentified victim in prior offenses, criminal conduct in the family, noncompliance with treatment, and a deficiency of adaptive coping mechanisms.
Grunwald et al. (2010) [44]	Social factor – parental criminality, neighborhood disadvantage	Parental criminality (*OR* = 1.27) and neighborhood disadvantage (*OR* = 1.19) significantly predicted recidivism, highlighting key social risk factors.
Castillo et al. (2010) [35]	Psychological factor – alcohol problem, bipolar disorder, comorbidity (bipolar × alcohol)	Alcohol problems was a strong psychological predictor of violent recidivism (Exp(B) = 9.828, *p* < .01), while bipolar disorder was only predictive when considered alone; comorbid conditions further emphasized the primary role of substance abuse. Compared to those without alcohol problems, individuals with alcohol problems were more likely to recidivate early and be arrested again for violent crimes.
Benner et al. (2010) [34]	Psychological factor – traumatic experiences, maltreatment, suicide ideation, depression/anxiety, anger/irritability	Trauma-related experiences (*ER* = 1.06), maltreatment (*ER* = 1.24), and alcohol/drug use (*ER* = 1.11) were significant psychological predictors of status offense recidivism among youth. Other clinical subscales, such as depression, suicide ideation, and anger, were included but were not statistically significant. Juveniles with a history of traumatic events, above-average alcohol and drug use, and maltreatment as children were four times (*OR*: 4.22) more likely to commit recurrent offenses while in juvenile status.
Ferguson et al. (2009) [46]	Psychological factor – criminal attitudes/orientations Social factor – criminal peers	Substance abusers were more likely to reoffend (59.8% vs. 38.6%, χ² = 11.19, *p* < .01) and had higher rates of risk factors, including criminal friends (41.3%) and attitudes supportive of crime (89.3%). Recidivism rates for mentally ill criminals who have been released from prison are probably going to drop dramatically if drug addiction issues are addressed.
LeBel et al. (2008) [5]	Psychological factor – hope/self-efficacy, regret and shame, internalizing stigma Social factor –relationships (partner/spouse and family)	In a 10-year follow-up, higher reentry problems (social factor) (*OR* = 1.38, *p* = .03) and elevated stigma (*OR* = 2.30, *p* = .07) were associated with recidivism, while positive identity (“family man,” *OR* = 0.49, *p* = .10) and hope (*OR* = 0.93, *p* = .08) showed protective trends. Any subjective criteria that predict the number of social difficulties experienced after four to six months may have an indirect influence on recidivism due to their impact on social problems. Reconviction and re-imprisonment were predicted by feelings of stigmatization (or being “doomed”), even after adjusting for the number of social issues that the individual had upon release.
Huebner et al. (2007) [48]	Psychological factor – proviolence attitudes, drug abuse Social factor – gang membership, community disadvantage, familial incarceration	Pro-violence attitudes (*HR* = 1.07, *p* < .01) and drug abuse (*HR* = 1.34, *p* < .10) were associated with higher recidivism (psychological factors); gang membership (*HR* = 1.90, *p* < .001), and community disadvantage (*HR* = 1.08, *p* < .10) were significant social predictors, while familial incarceration was not.
Kubrin and Stewart (2006) [45]	Social factor – disadvantaged neighborhoods	Recidivism risk varies significantly across neighborhoods (*p* < .01), with risks ranging from less than 5% to over 40%, suggesting that neighborhood-level social factors influence recidivism. Property, drug, and “other crime” (as opposed to violent) incarcerated individuals, as well as those who obtained additional criminal penalties, may have greater recidivism rates under intensive supervision. Due to restricted access to resources, support networks, and chances for rehabilitation and reintegration into society, living in a community marked by poverty, inequality, and socioeconomic disadvantage might raise the likelihood of recidivism.
Hepburn (2005) [38]	Psychological factor – poor therapeutic engagement	Individuals with addictions with greater recidivism rates are frequently those who refuse or put off getting involved in treatment programs. Their resistance might be due to psychological problems like denial about how serious their addiction is or misbelief in the efficacy of the therapy, which can lead to a lack of willingness to change.
Långström et al. (2004) [39]	Psychological factor – psychiatric disorders	Psychiatric conditions such as personality disorders (*OR* = 10.1), psychosis (*OR* = 5.1), and substance use disorders (*OR* = 2.3–2.9) significantly increased the odds of sexual and violent nonsexual recidivism. While personality disorders and alcohol use disorders predicted violent nonsexual recidivism, drug use disorders, psychosis, and alcohol use disorders all raised the chance of sexual recidivism. A recent rape conviction was linked to an increased chance of violent, but not sexual, recidivism. These disorders’ underlying behavioral and psychological problems impede judgment, heighten impulsivity, and weaken the capacity to restrain violent or deviant behavior.
Gagliardi et al. (2004) [37]	Psychological factor – mental illness	Among mentally ill offenders, 69% were reconvicted and 10% committed a new violent felony over 27–55 months — comparable to general population offenders — while past drug felonies (*r* = .35), total felony history (*r* = .40), and offense versatility (*r* = .31) were also significant predictors of recidivism. Recidivism is not higher for mentally ill prisoners than it is for those who are well.

### Psychological factors influencing recidivism risk among incarcerated individuals with a history of substance use

Psychological dysfunction was consistently linked to an increased risk of recidivism across studies, particularly when it co-occurred with substance use disorders (SUD). Seven studies found that mental illnesses, such as mood disorders, significantly contribute to reoffending, especially when untreated [33,35–39,42]. Three of those studies discovered that the impact was enhanced by co-occurring SUD, and that dual diagnoses were linked to a greater risk than either condition alone [35,36,42]. Particularly, individuals with bipolar disorder were identified as high-risk because of their impulsivity and mental instability, which were made worse by substance use [35,36].

Additionally, two studies found that recidivism is predicted by mentally ill individuals with certain psychological diagnoses, such as personality disorders and psychosis, particularly among violent or sexual offenders [37,39]. These disruptions were frequently linked to increased impulsivity, poor coping, and trouble regulating emotions. Incarcerated individuals who are mentally ill tended to exhibit different patterns of conduct, even in cases when overall reoffending rates were the same [37]. Stahler et al. [41] corroborated these results by demonstrating that substance usage by itself raised the risk of reincarceration by about 40%. In a similar vein, Ojansuu et al. [40] discovered that during a follow-up period of five to fifteen years, incarcerated individuals with SUD had a reoffending rate that was more than twice as high. Notably, over half of these recurrent misconducts were violent, where people with untreated psychological and substance-related conditions exhibit persistent behavioral instability.

This risk was further increased by unmet psychological needs during reentry. Begun et al. [33] found a significant discrepancy between those who received services to address their psychological problems (15%) and those who anticipated post-release mental health needs (56.5%). Hepburn [38] also noted psychological barriers like low motivation or denial that decreased engagement even in the presence of services.

Lastly, it was shown that early psychological stress was also an essential component. Two studies [34,35] found that trauma and abuse throughout childhood were associated with a higher likelihood of committing crimes later on, especially when drug use was present. While symptoms such as depression or suicidal thoughts did not predict reoffending on their own, Benner et al. [34] discovered that those with a history of abuse had a higher likelihood of being arrested as adults (42%), compared to youth (27%), where a delayed but long-lasting psychological impact contributes to adult reoffending.

### Social factors influencing recidivism risk among incarcerated individuals with a history of substance use

Social determinants such as familial support, parental criminality, and neighborhood disadvantage were consistently associated with recidivism risk. A protective element across studies was family emotional support, particularly during the sensitive reintegration phase. Higher levels of emotional support from family members were linked to lower recidivism rates and longer time between incarcerations, according to three studies [5,43,49]. Two of them discovered that the protective influence of family relationships and support networks offered emotional stability and lessened dependence on unhealthy coping strategies [43,49]. Support alone, however, was not always sufficient, as demonstrated by the findings of Barrick et al. [43]. Even among individuals with strong emotional ties, reoffending happened when additional risk factors, including socioeconomic instability or unresolved psychological difficulties, went untreated [50].

Conversely, unfavorable social circumstances were linked to a higher probability of recidivism. According to two study [44,45], those who were exposed to parental criminality or structural poverty at the community level had a higher likelihood of committing crimes again. These environments frequently reduced possibilities for employment or community reintegration, normalized illicit behaviors, and weakened access to prosocial role models. Specifically, parental criminality and living in a socially poor neighborhood were found to be important predictors of juvenile reoffending by Grunwald et al. [44]. Similarly, Kubrin and Stewart [45] found that rearrest rates differed significantly amongst neighborhoods, with greater rates concentrated in areas with poor social cohesiveness and inadequate access to support systems.

### Interplay between psychological vulnerabilities and social environment

Research repeatedly shows that psychological and social elements do not work in isolation, but rather frequently combine in intricate ways to influence incarcerated individuals’ possibility of reoffending. Across the studies reviewed, both psychological and social factors were consistently linked to recidivism risk among those with a history of substance use. Substance use has been shown to dramatically increase the risk of recidivism in four studies [54,56–58], especially when paired with exposure to unstable environments, criminal peer associations, or inadequate family support. For instance, one of these studies found that violent repeat offending was much more common among incarcerated individuals who had substance use issues and troubled social relationships [54]. Another research [58] found that high-risk people had greater rates of drug issues and were more likely to originate from single-parent families or non-traditional family structures.

Five studies [46,48,53,54,60] evident that social risks often exacerbate psychological vulnerabilities, including pro-criminal attitudes, poor self-control, mental illness, or personality pathology. For example, two studies highlighted the importance of gang membership and mental health decline as reinforcing processes, wherein societal and psychological elements contributed to persistent criminal conduct [48,60]. One study [53] discovered that incarcerated individuals with antisocial or emotionally dysregulated traits were more likely to experience repeated misconduct issues and interpersonal conflict within institutions, to which psychosocial maladjustment perpetuates both in-prison and post-release risk.

Furthermore, three studies [49,50,60] showed that untreated mental illness, specifically depression, psychiatric instability, and emotional dysregulation, was a significant predictor of recidivism when it co-occurred with criminogenic networks, family conflict, or financial stress. One of this research [50] discovered that depressive symptoms indirectly enhanced reoffending through higher social strain, whilst two others revealed that psychological changes and emotional support after release might reduce these risks [49,60].

Four research [4,55,59,60] identified social instability, including homelessness, inadequate parenting, and disturbed peer or family connections, as a risk factor. In Karlsson and Håkansson’s [4] study, homelessness, in particular, stood out as a key predictor. When coupled with mental health issues, its influence was exacerbated, further solidifying the psychosocial vulnerability factor. The remaining research showed that exposure to peer rejection, familial dysfunction, or disengagement from school frequently happened in conjunction with internal vulnerabilities, including maladaptive attitudes, poor impulse control, or mental illness, increasing the likelihood of violent reoffending.

Three studies [5,49,59] showed that recidivism rates were decreased by solid family or partner ties, systematic supervision, emotional support, and a positive sense of self. One longitudinal research [5], for instance, discovered that those who preserved strong familial relationships and supported prosocial identities had a higher chance of being conviction-free for ten years.

Additionally, the three qualitative studies [47,51,52] highlighted that substance use was not only a personal habit but rather a component of a larger psychosocial path to identity creation, emotional regulation, and survival in challenging situations. Two studies [47,51] found that emotional discomfort, portrayed as guilt, despair, and poor motivation, was frequently exacerbated by social isolation, character contamination from the cell population, and a lack of relationship support. In both situations, a lack of prosocial options and a lack of interest in recovery led to substance use as a coping strategy. One study [51] found that post-release problems such as stigma, rejection, and a lack of social scaffolding enhanced emotional distress. In Mohd Alif et al.’s [52] study, substance use was depicted as symbolically meaningful, where it provided a sense of control and belonging in the face of persistent external constraints such as poverty, peer normalization of crime, and unmet mental health needs.

## Discussion

The purpose of this systematic review was to investigate the interaction between psychological and social variables and their influence on recidivism risk among people with a history of substance use and imprisonment. Additionally, the objective was to identify the primary psychological and social determinants that serve as risk factors in order to comprehend how their interaction influences the outcomes of reoffending. The findings imply that psychological and social elements work together to increase the probability of recidivism, with various studies highlighting their impact.

The findings of this review contribute to our understanding of recidivism among individuals with a history of substance use and incarceration by demonstrating how psychological and social dimensions interact to determine reoffending risk. Instead of considering these domains as distinct, the synthesized data points to a more dynamic, integrated model in which criminal trajectories are co-produced by vulnerabilities at both levels. The reinforcing loop between substance use and mental health issues, especially when they occur in unstable social environments, is one noteworthy finding from this synthesis. According to earlier studies, substance use frequently serves as a coping strategy when confronted with trauma or mental distress [61,62]. This may help explain why substance use persists after incarceration, even though there are treatment programs available that might not fully address these underlying psychological needs. This is particularly pertinent since long-term use of substances has been demonstrated to change brain networks related to impulse control, stress management, and reward processing [63]. These neuroadaptations might hinder executive functioning and diminish an individual’s capacity to control their emotions or delay gratification, particularly when faced with psychosocial stresses. Hence, these brain alterations may increase an individual’s susceptibility to repeated and impulsive behaviors when they return to contexts that are characterized by instability, a lack of social support, or exposure to cues from past trauma. For example, those who were returning to settings without family support or access to integrated treatment frequently had untreated mental health symptoms and substance dependence [64]. These factors are believed to exacerbate discomfort, worsen emotional dysregulation, and jeopardize psychological stability. In many situations, the resumption of substance use seems to be a reaction to escalating stresses, such as disruptive emotions and social isolation, rather than a personal decision.

Despite the fact that mental illness has long been recognized as a risk factor for recidivism, especially for those with past substance use, the results of this review support more recent research that indicates psychiatric diagnoses alone do not significantly predict reoffending [53,60]. Rather, the research backs up the idea that mental illness becomes criminogenically relevant when it is entwined with other psychosocial vulnerabilities, such as trauma histories, criminal convictions between family members, or a deviant network that encourages reoffending. This builds on Walters’ [65] notion that the relationship between mental illness and recidivism is frequently indirect and dependent on mediating variables rather than innate pathology. For example, those who have family problems could find it difficult to adjust to reintegration in situations where they are exposed to criminal networks or socially unorganized. In such situations, disturbed family relations or a lack of emotional support may hinder an individual’s capacity to cope with stress, control emotions, and make adaptive decisions; factors that, taken together, increase vulnerability to relapse and reoffending [66]. Such settings have the potential to worsen underlying psychological disorders, hence increasing the probability of reoffending through a cyclical process. Notably, this reframe encourages a shift from deficit-focused perspectives that regard recurrence as the outcome of poor compliance or resistance to treatment. Instead, recurrence may indicate a failure of context, in which reintegration lacks the stability, relational support, or trauma-informed scaffolding required to maintain recovery [67,68]. This explains why ostensibly protective variables, such as familial relationships, can often increase risk: when these connections are unstable or difficult, they may compound rather than buffer psychological strain [5].

The protective effect of social and familial support in lowering the likelihood of recidivism among individuals with past substance use histories who have served time in prison is supported by two studies. Emotional support was shown to delay time to recidivism and reduce probabilities by around 19%, indicating that those with greater levels of post-release family emotional support were considerably less likely to reoffend [43]. Similarly, during a ten-year study, LeBel et al. [5] found that preserving a positive family identity, such as considering oneself a family man, was associated with a lower likelihood of reoffending. Their results indicate a potentially neglected leverage point in post-release interventions: family support may promote pro-social identity and serve as a buffer against psychological strain.

Although family support is mentioned as a stabilizing component in desistance [69], a number of studies in this review indicate that its protective function can depend on other elements, including psychological stability. Individuals returning to families with histories of conflict, dependency on substances, or mistreatment reported minimal benefit or even increased stress [13,59]. These results imply that rather than presuming consistently favorable results, reentry programs incorporating family participation should be considerate of individual readiness, familial dynamics, and mental health requirements. Comparably, even though peer influence and neighborhood disadvantage are well-established risk factors for recidivism [45,70], this review supports evidence that socially disorganized or high-risk environments, especially those devoid of positive peer role models, may perpetuate psychosocial vulnerabilities and impair reentry outcomes. In these situations, peer networks frequently catalyze relapse and reoffending rather than as a protective barrier, particularly when they are not included in organized, pro-social support networks.

These findings highlight the need for multi-level, coordinated approaches that target both more general psychological readiness and social weaknesses on an individual basis. The findings demonstrated how involvement in community-based treatments, post-release supervision, and pre-release planning had major advantages. Research in the field of rehabilitation strongly supports the claims that individuals who participate in organized reintegration initiatives have lower reoffending rates than those who do not receive institutional support [71,72]. Additionally, the current systematic review noted that several programs have been successful in lowering criminal recidivism, including community notification systems for high-risk people with a history of imprisonment and forensic mental therapy [40]. This finding supports the literature’s reports that intense, personalized therapies show stronger positive effects than generic rehabilitation programs in terms of long-term results [73,74]. However, the findings of this review indicate that differences in program availability and execution continue to be an issue since not all individuals have equal access to these rehabilitative initiatives.

### Limitations and potentialities

While this review synthesizes significant psychosocial determinants of recidivism, some limitations warrant acknowledgment. One major drawback of this review is the variability of the research populations, notably in terms of gender and age. Several studies focused solely on adult male populations, while others included juvenile offenders or female participants, frequently without breaking down results by subgroup. This heterogeneity makes it challenging to draw generalizations across different demographic groups since the psychological and social variables that influence recidivism may show up differently in males and females or adolescents and adults. Furthermore, the operationalization of recidivism was inconsistent. Most research used administrative data, such as arrest records, reconviction rates, or re-incarceration, as proxies for recidivism. Although they are widely used indicators, they cannot not adequately account for the complexities of recidivism, including changes in the judicial system or unreported criminal activities. Direct comparisons are made more difficult by the variation in sample composition and outcome measurement. Moreover, the variation in recidivism categories among the included studies adds complexity to the analysis, since different crimes may include various risks. However, this is a deliberate methodological decision rather than a constraint, since it allows for a more thorough understanding of the common psychosocial pathways that contribute to recidivism. This more comprehensive approach is consistent with data indicating that criminogenic demands are developed by social and psychological processes, such as substance use patterns and psychosocial factors, rather than being intrinsic.

While some studies concentrated only on sexual recidivism, all of them included background information on substance use, which adds complexity to the analysis of recidivism and makes it challenging to compare results across different kinds of offenses. Furthermore, there were notable differences in the duration of time at risk for recidivism amongst studies, ranging from brief to extended follow-ups. Though this might potentially point to possible biases in reporting or measurement, the results frequently converged despite this heterogeneity, showing some stability in recidivism patterns. While cross-sectional data is useful for providing a quick overview, it restricts the ability to draw conclusions about the long-term interactions between social and psychological elements. Even though the review contains research from a variety of geographic and cultural contexts, this diversity both enhances our understanding of the subject and draws attention to the difficulties associated with generalizing results to other groups. As research with more noticeable findings is more likely to be published, publication bias is a factor that should be taken into account, as it might affect the conclusions that are made overall. Despite these drawbacks, the analysis provides a strong basis for future study and useful application in the area by synthesizing a wide variety of information and identifying significant factors impacting recidivism.

While this systematic review synthesizes data from research on various crimes, it is essential to note that their fundamental basis lies in shared psychosocial components. The current review emphasizes how psychosocial variables, including family support, economic stability, mental health, trauma, and drug use, consistently shape recidivism risk, even in the face of variations in legal classifications and particular criminal typologies. This convergence implies that interventions aimed at these variables may not only affect certain crime categories but also have a wide-ranging effect on recently released adults. One of this review’s main potentialities is that it supports the idea that criminogenic demands are socially acquired and reinforced throughout time rather than being innate characteristics. This review highlights how long-term social vulnerabilities, including substance use, mental health treatment inaccessibility, and marginalization, progressively translate into psychological functioning that perpetuates criminal cycles. Specifically, as a maladaptive coping strategy influenced by larger psychosocial stresses, substance use appears as both a cause and an effect of criminal activity. This analysis emphasizes the premise that successful recidivism prevention requires addressing societal factors in addition to individual rehabilitation efforts.

### Implications

The findings of this analysis have important implications for both practice and policy in the criminal justice and public health sectors. The necessity for treatment options that address both the specific psychological needs and their wider social milieu is highlighted by the substantial correlation shown between psychological issues, such as maladaptive coping strategies, and social factors, such as peer influence. For example, social support interventions that focus on peer connections and community reintegration might be incorporated with cognitive behavioral therapy programs designed for people with a history of substance use. The research also points to the necessity of continuity of care for people with a history of imprisonment when they are released from prison, with a focus on ensuring that they have access to social support networks, drug treatment centers, and mental health initiatives that reduce their risk of recidivism.

### Future research

Given the identified gaps in the literature, future research should focus on investigating the interplay of social and psychological components in a variety of cultural and socioeconomic circumstances. Future research ought to emphasize addressing the issues raised in this article while exploring areas that have not been investigated. Longitudinal studies, which follow incarcerated individuals over protracted periods and offer a more thorough knowledge of how psychological and social variables impact recidivism over time, are one important path. Individuals with a history of imprisonment may be hesitant to commit to long-term engagement. To overcome these constraints, future research should look into alternate methodologies, such as employing administrative longitudinal datasets or combining mixed-methods designs with periodic follow-ups that respect participant autonomy. Using peer researchers and forming community-based collaborations may help improve retention and trust in these investigations. This method may make it easier to understand the causal relationships and temporal dynamics between psychosocial factors impacting the risk of recidivism among people with a history of substance use and incarceration. Future research should also aim to unify recidivism definitions and metrics while taking into account the biases caused by different kinds of crimes and time-at-risk.

Future research should consider stratified analyses to better capture population-specific patterns and risk profiles. Future reviews might benefit from taking a more intersectional approach, looking at the ways in which psychological and social characteristics interact with variables like race, gender, and socioeconomic position to impact recidivism. Furthermore, research comparing the efficacy of various intervention techniques is needed to determine the best methods for lowering recidivism among people with a history of substance use. This is especially relevant for interventions that combine psychological treatment with social support networks. Future research can help establish more focused and efficient treatments by filling these gaps, which will eventually increase the effectiveness of rehabilitation programs and lower recidivism rates.

## Conclusion

The findings of this review contribute to our comprehension of recidivism among individuals with a history of substance use and incarceration by demonstrating how psychosocial dimensions interact to determine reoffending risk. Instead of considering these domains separate, this review points to a more dynamic, integrated model in which criminal trajectories are co-produced by vulnerabilities at both levels. The incorporation of psychological and social elements provides a more comprehensive understanding of recidivism, emphasizing the significance of treating psychological as well as social aspects in rehabilitation initiatives. While the study finds significant trends across studies, it also recognizes the limits of current research, such as the variety in recidivism definitions and gender-specific studies, which may introduce biases into results. The analysis underscores the importance of tailored treatment options that cater to the unique requirements of substance users and integrate psychological and social care, even in the face of these challenges. The implications for practice and policy are significant, implying that lowering recidivism rates requires an all-encompassing approach to rehabilitation. Future studies should focus on filling the gaps found in this study, especially by using longitudinal research and standardized recidivism metrics. By doing this, the profession may advance toward more potent techniques for halting the cycle of reoffending, which will eventually improve outcomes and community safety.

## Supprting information

S1 Appendix Prisma 2020 Checklist. (DOCX)

S1 File Quality Assessment. (XLSX)
